# 

**Published:** 1899-01

**Authors:** 


### CONTENTS.

COMMUNICATIONS.

Lecture with Stereoptican—Dental Caries; Its Character, Cause and Effects ........ 1
Dental Schools Abroad........ 11
Notes on Pyorrhea Alveolaris ........ 17
New Treatment for Exposed Tooth-Pulp........ 31

PROCEEDINGS.

New Ohio State Dental Law ........ 36
Resolutions ........ 40

SELECTIONS.

What Hypodermatic Tablets Ought To Be ........ 41

NOTICES.

Second Annual Meeting of the Southern Branch of the National Dental Association ........ 42
Chicago Dental Society ........ 43

EDITORIAL.

War Against Bogus Colleges and Degrees ........ 43
Dentists in the Army and Navy........ 45
Who Shall Prepare Our Food? ........ 47
Directory for Dental Societies and Colleges ........ 48

OBITUARY.

Dr. J. B. Hamilton ........ 49

